# What is hidden in hidden curriculum? a qualitative study in medicine

**DOI:** 10.18502/jmehm.v13i4.2843

**Published:** 2020-05-10

**Authors:** Shahram Yazdani, Mohammad Reza Andarvazh, Leila Afshar

**Affiliations:** 1 *Professor, School of Medical Education Sciences, Shahid Beheshti University of Medical Sciences, Tehran, Iran. *; 2 *PhD Graduate of Medical Education,* *School of Medical Education, Shahid Beheshti University of Medical Sciences, Tehran, Iran; Assistant Professor, Nasibeh School of Nursing and Midwifery, Educational Development Center, Mazandaran University of Medical Sciences, Sari, Iran. *; 3 *Associate Professor, Department of Medical Ethics, Shahid Beheshti University of Medical Sciences, Tehran, Iran.*

**Keywords:** Hidden curriculum, Medical education, Professional ethics, Implicit learning, Content analysis

## Abstract

The hidden curriculum is considered to be between the designed and experienced curricula. One of the challenges that medical educators face is to understand what students learn in real clinical settings. The aim of the present study was to answer this question: What is hidden in hidden medical curriculum?

This study was a qualitative content analysis. Participants were selected through purposive sampling. Data collection was performed through unstructured interviews and continued until data saturation.

Data were analyzed simultaneously with data collection using MAXQDA10 software. Data validity was confirmed based on the proposed Lincoln and Guba criteria.

The main theme that emerged in this study was implicit learning. Professional ethics, spiritual, social and cultural issues, and clinical skills are the five major themes that were presented in this study. These themes and their subthemes are transferred during an implicit learning experience in hidden curriculum.

Since a wide range of issues are mostly transferred by hidden curriculum, it is essential to have a dynamic approach to educational environments. This is especially important in clinical settings, as the process of learning is constantly happening in the backyard.

## Introduction

Two decades after the introduction of hidden curriculum by Philip Jackson in 1968, Hafferty put this concept forward in medical education (1). He stated that hidden curriculum is responsible for most of the material that is learned in medical schools(2).

Analyzing hidden curriculum is neither simple nor without challenge. What is directly understood from such an analysis might be odd and shocking or inconsistent with common sense(3).

Medical students learn many key and important issues from hidden and informal curriculum and not from formal medical curriculum(4). In the recent years, curriculum is no longer a term that clearly incorporates formal and genuine documents into all planned and unplanned experiences conducted under the supervision of an educational institution(5).Much of what educational systems teach is planned and explicit, but there are topics that are implicit, unplanned and hidden, and can be found in hidden curriculum(6).

Some of the learners’ attitudes and behaviors are shaped by hidden curriculum in an institution’s learning environments(4).Economically, hidden curricula impose a lot of health care costs on the society(5). In medical science education, hidden curriculum has the most impacting clinical education settings(7). It has a very powerful influence on the formation of students' professional development and may change their general ideas about career and clinical practice (8). In Iran, educational researchers have not neglected studies of hidden curriculum and have added to the literature through their research results(9).Most of this research, however, has been done in middle and high schools, and a small part pertains to higher education, especially in the medical education system.

Considering the importance of hidden curriculum in medical education, especially clinical education, the present study was conducted. The aim of this study was to analyze the content of hidden curriculum in order to determine what it conveys to students in clinical educational environments. The study was a qualitative inquiry based on students’ and faculty members’ experiences and aimed to grasp their viewpoints on the issue.

## Methods

This research was a content analysis designed to help understand the concepts in qualitative analysis(10).What made it suitable for qualitative research was the fact that it provided clear evidence of a phenomenon(11). Content analysis has been described as a qualitative study method for subjective interpretation of text data by means of systematic categorization of coding and determining patterns and themes (12).

Eighteen faculty members and medical students voluntarily participated in the study. For maximum variation, they were recruited by purposive sampling from different specialties and levels. The study was conducted between May 2016 and February 2017. In terms of inclusion criteria, the faculty members had to have a minimum of five years’ work experience as clinical teachers in medical schools, and the medical students were selected from undergraduate and postgraduate levels in the general clinical fields. 

Data were collected through unstructured interviews. The main questions included: “What do students learn in clinical settings?” (asked from teachers), and “What do you learn in clinical settings?” (asked from students). The probe question was “What do you learn in clinical settings apart from formal curriculum? “Each Interview lasted 45 - 120 minutes. Interviews were transcribed verbatim immediately after being recorded and were then analyzed. The interviewer had formal qualitative curriculum education and several years of experience in academic teaching, which would help in establishing trustworthy communication. The interviewer did not have any relation to the interviewees. Participants’ selection, data collection and analysis continued until data saturation and a rich explanation of participants’ experiences were achieved. Data collection was completed after 22 interviews (four participants were interviewed two times), when no new concepts emerged.

Five steps of "framework" process in the conventional content analysis approach were utilized to analyze data in this study.(10). The first step was familiarization, in which the audiotape recordings were transcribed word forward. The authors began data analysis immediately after the first interview. To get a sense of the whole subject, the transcribed interviews were read several times. In the second step, or thematic framework identification, the researchers analyzed the data word by word and line by line to identify the meaning units in order to determine the important concepts, themes and issues. Next, they abstracted reduced meaning units and coded them top resent the content. In the third step, the various codes were compared based on differences and similarities, and the emerged codes were sorted into subcategories and categories. The fourth step consisted of applying the thematic framework to individual transcripts and making a picture of the data. Collected extracts for each category were read by the authors to form a coherent pattern. Then, the validity of each category was evaluated in connection to the data set and selected categories. Two researchers examined the data for the categories independently. In the fifth step, or mapping and interpretation, the categories were defined and further refined. We used MAXQDA 2010 software to facilitate analyzing the data. 

The criteria of Guba (1981) were applied in this study to ensure trustworthiness(13). Credibility was achieved through variation in participants’ work experience, gender, type of specialty and grade of education among the faculty members, and type of specialty, area and grade of education and gender among the students, to get an extensive explanation of the phenomena. The main researcher used field notes to increase data quality and had prolonged engagement with the study fields. For peer checking, five experts with PhD degrees or medical specialties controlled and confirmed the coding and categorization process. All the peer checkers were faculty members with considerable experience in performing and teaching qualitative studies. For member checking, the abstracts of the interviews were returned to the participants and they verified the results.

The Research Committee of Shahid Beheshti University of Medical Sciences issued the ethical approval for the study protocol (No. 130, Date: 26/10/2015). The nature and aim of the study were explained to the participants, and their verbal and written consent were obtained. Furthermore, study subjects were assured of confidentiality and voluntariness of participation.

## Results

Ten faculty members (3 females and 7 male) and eight students (4 females and 4 male) were interviewed (Table 1). 

**Table1:** Participants’ demographics

Number	Gender	Area of Practice	Age (Years)	Academic Degree	Work Experience (Years)
1	Female	Cardiology	45	Associate professor	13
2	Male	Psychiatry	57	Professor	26
3	Male	Anesthesiology	47	Professor	20
4	Male	Emergency medicine	47	Assistant professor	13
5	Male	Rheumatology	52	Associate professor	23
6	Female	Psychiatry	42	Associate professor	10
7	Male	Immunology	43	Assistant professor	7
8	Female	Pathology	45	Assistant professor	10
9	Male	Orthopedics	51	Associate professor	17
10	Male	Internal medicine	48	Assistant professor	12
11	Female	Emergency medicine	31	Student	Not applicable
12	Male	Internal medicine	46	Student	Not applicable
13	Male	Internal medicine	38	Student	Not applicable
14	Female	Anesthesiology	32	Student	Not applicable
15	Female	Undergraduate	23	Student	Not applicable
16	Male	Undergraduate	24	Student	Not applicable
17	Female	Undergraduate	24	Student	Not applicable
18	Male	Undergraduate	25	Student	Not applicable

Data analysis resulted in 119 open codes from which 13 sub-themes and 5 themes were derived. The five themes consisted of professional ethics, spiritual, social, cultural and clinical skills, with subcategories. Implicit learning was recognized as a main theme (Table 2).

**Table2:** Main theme, themes and sub-themes of implicit learning through hidden curriculum

Main Theme	Themes	Sub-Themes
Implicit learning	Professional ethics	Educational ethics
		Research ethics
		Clinical ethics
	Spiritual issues	Belief in the hereafter
		Lack of belief in the hereafter
	Social issues	Career path
		Hierarchy
		Teamwork
		Lifestyle
	Clinical skills	Correct medical procedures
		Incorrect medical procedures
	Cultural issues	Intra-organizational culture
		Extra-organizational culture

The main theme derived from the whole interview was implicit learning, which consisted of 5 categories including professional ethics, spiritual, social and cultural issues and clinical skills, with subcategories. Below, the participants' statements about themes and subthemes are explained.

***1) Professional Ethics***

In this theme the participants provided examples that were then put under the sub-themes educational, research and clinical ethics. The message in each theme demonstrate the perceived degree of ethical importance of the issue.

The following are some of our participants’ statements:

*"One of my teachers was very rude and did not respect any of the students or other teachers. Most of his trainees imitated this type of behavior. The fellows who are now working or have worked under him have acquired his rude behavior.” *[Participant No. 12]

*"If student is doing dissertation with me, I supervise exactly what he/she is doing, and we work together. After he/she completes the work, I ask for the sources of information, the file numbers and everything, and then all the information and data are entered into SPSS under my supervision or that of an expert. In this manner, the student realizes that working accurately is an important issue in research."*[Participant No. 1]

"Teaching medical ethics should not be the way it is commonly done, it’s not like writing a book or making a lecture. These are things that really need to be taught through teachers’ behaviors, and behavior is not verbal, it is non-verbal." [Participant No. 2]

***2) Spiritual Issues***

The participants stated that spiritual issues could be transferred through hidden curriculum. This theme is divided into two parts: belief in the hereafter, and lack of belief in the hereafter.

The participants’ statements in this regard were as follows: 

*"* *Belief in the hereafter affects teacher’s behavior, and this behavior has the highest influence on students. Whether or not a teacher has religious or spiritual beliefs can be influential."*[Participant No. 2] 

*"If I do not believe in God and do not consider His consent in my work, it will affect my relationship with the patient; I will measure everything according to money, and my students will see and learn." *[Participant No. 9]

***3) Social Issues***

The results of implicit learning can be found in social matters and life in the social environments of a hospital, or even outside of it. Learning about issues such as career path, hierarchy, teamwork and lifestyle occurs via hidden curriculum in clinical situations.

In this regard some participants said:

*"Students compare my financial status - as a specialist in a discipline - with another colleague who has another specialty, and see that I have a cheap car, but he has an expensive car. They conclude that they should choose a field that ensures better financial position. This has not been taught to the students, but they draw conclusions*”. [Participant No. 5] 

*"When we see students walking at a distance behind the teacher, the message is that the position of the professor is much higher than the students."*[Participant No. 6]

*"The routine in the medical profession is that we work as team members. I always try to visit my patients with a nurse and a psychologist. It’s not that they are with me, but their opinion is important to me. My professors did this and I learned from them." *[Participant No. 6]

*"Students are psychologically very capable of modeling. They are not hereto just attending a medical course; they see their teachers and learn from them life lessons." *[Participant No. 7]

***4) Clinical Skills***

It is said that many techniques are learnt implicitly. This learning can be right if students see the right methods in the department, and wrong if they see wrong ones.

Participants stated**: **

*"We learn a lot about interviewing patients. For instance, nobody teaches us not to laugh at patients and their company; also, we learn what to do and how to ask questions so we won’t annoy the patients and obtain their history in order to treat them better. Although the professors give us a brief academic description of the process, they don’t tell us how to do it. We learn all this in departments."*[Participant No. 18]

*"Spoke for many years about ethics and techniques: You have to stick to sterilization... you should wear gloves… bring the book and teach it and then do not attend the patient’s bedside. If I put the CVP line for a patient without wearing gloves, forty years of training collapses and disappears." *[Participant No. 3]

***5) Cultural Issues***

Culture is one of the issues transmitted by hidden curriculum in the hospital environment, and can be divided into intra-organizational culture and extra-organizational culture.

The following are some statements made by our participants:

*"All professors have social, cultural and technical characteristics beside their medical characteristics. You can find it absolutely anywhere, even in the best centers you can find mean people, you can see both mean and kind attending doctors, organized or disorganized ones. All of these people together determine the potentiality of the center’s culture and its outcome. These things are clear…"*[Participant No. 3]

*"Patients and their family, even colleagues, bring the outside world culture into the hospital. We learn a lot about the cultures of the external community from them*."[Participant No. 11]

## Discussion

The present study was based on a qualitative inquiry and showed that the content of hidden curriculum could be categorized as: professional ethics, spiritual issues, social issues, clinical skills and cultural issues. This categorization would help educators and educational organizations to better manage the effects of hidden curriculum.

As mentioned earlier, the first theme of the present study was one of the consequences of hidden curriculum, which is transferring professional ethics. There is an agreement between educators that hidden curriculum has a significant role in shaping the professional identity of students (14). In line with the present study, Jafree et al asserted that medical trainers, instructors and senior licensed practitioners influence the future of the moral behavior of their students and colleagues through hidden curriculum(15).

Olthuis and Dekkers stated that medical training is a process of moral enculturation in the medical community. This process is not often explained clearly in formal curriculum(16).

In transferring norms, rules and beliefs, As Kently quoted; Henry Siouxs referred to hidden curriculum as the norms, values and beliefs that are embedded in it and are transferred to students through regulations, structures and social relationship in schools and classes(17).

Concerning the transfer of professional values, Thiedke et al. asserted that most professional values are conveyed through hidden or informal curriculum, a social setting that is broader than medical training, and lies outside of formal teaching(18).

Many authors declared hidden curriculum is declared that hidden curriculum is the impalpable informal and implicit teaching of values, norms and attitudes that are created and transferred by the medium of instructors, practitioners, informal teaching processes, students’ interactions and their interpretation of organizational events and environments(1, 2, 16, 19).

Stanek et al. stated that hidden curriculum indirectly alters the interactions, beliefs and practices of medical practitioners during their education(19). In addition, some studies showed that values, norms and belief systems are transmitted through hidden curriculum(17, 19).

Concerning the significance of professional behavior in the professional development of students, John ston et al. found that observing teachers acting un-professionally has a potential damaging impact on the professional development of students. The negative consequence of hidden curriculum arises when there is a contradiction between students' feelings and what they need to do in order to conform to the medical culture. This can lead to failure to address complicated moral occasions appropriately, despite having the required knowledge(21). Azmand et al. concluded in their study that hidden curriculum has an effect on the development of the present “ethical climate”, by which medical students’ professional and ethical identities are shaped. In addition, specific plans regarding the institutional settings may provide opportunities to increase professionalism in institutions for medical educators(22).

The second theme that was explored in the present study was spiritual issues. In line with our study, Balboniet al. stated that religion/spirituality possibly has an effective role in the socialization of medical students, but has been largely unstudied. Therefore, further research is needed to find its effect on hidden curriculum(23).

The third theme in the present study was social issues. As quoted by other researchers, Jackson said that students must learn to demonstrate appropriate social abilities(24). According to Hashemiet al. social skills are transferred through hidden curriculum (25).

The fourth theme in the present study was clinical skills. Rojas stated that skills are learned through hidden curriculum during the clinical practice period(26). Moreover, some studies indicated that practices (24) and behaviors (27) are transferred by hidden curriculum. 

The fifth theme was cultural issues. Wear and Skill corn stated that hidden curriculum includes a set of unwritten rules, social and cultural values, expectations and assumptions, and is more influential in comparison with formal curriculum(5). Besides, some studies asserted that cultural norms and customs(28) and social and cultural values, rules, and assumptions(29) are transmitted by hidden curriculum.

## Notes:

**Citation to this article:**

Yazdani S, Andarvazh MR, Afshar L. What is hidden in hidden curriculum? a qualitative study in medicine. J Med Ethics Hist Med. 2020; 13: 4.
